# The cardiology community begins to embrace obesity as an important target for cardiovascular health

**DOI:** 10.1371/journal.pmed.1004578

**Published:** 2025-04-24

**Authors:** Naveed Sattar, Martin K. Rutter

**Affiliations:** 1 School of Cardiovascular and Metabolic Health, University of Glasgow, Glasgow, United Kingdom; 2 Division of Diabetes, Endocrinology and Gastroenterology, School of Medical Sciences, University of Manchester, Manchester, United Kingdom; 3 Diabetes, Endocrinology and Metabolism Centre, Manchester University NHS Foundation Trust, Manchester Academic Health Science Centre, Manchester, United Kingdom

## Abstract

Naveed Sattar and Martin K Rutter discuss the contributory role of obesity in the development and progression of cardiovascular disease, and prospects for tackling the obesity epidemic.

Despite declining incidence of atherosclerotic outcomes, non-atherosclerotic outcomes and multimorbidity (both linked to obesity) are rising in cardiology clinics, stretching services. This article discusses these issues and suggests potential ways forward.

Recent cardiovascular meetings worldwide have seen a noticeable increase in content focused on obesity. This shift reflects a growing recognition of the role of obesity in cardiovascular disease alongside the major atherosclerosis risk factors, namely lipids, blood pressure and smoking. For decades, prevention efforts have focussed on these established risk factors, capitalising on the availability of proven interventions, developed through dedicated clinical trials. Consequently, over the past three decades, increasing numbers of people have received statins and blood pressure medications, initially in secondary and then in primary prevention settings. At the same time, population-level smoking rates have declined. From the 1990s to the present day, this focus on traditional risk factors has translated into rapid declines in incident atherosclerotic cardiovascular disease (ASCVD) events in high-income countries like the UK [1], though data in recent years suggest a plateau. While the impacts of the COVID-19 pandemic are yet to be fully understood, fewer people are now dying from premature cardiovascular disease, which has contributed to rising life expectancy. While these achievements are to be celebrated, there remains room for improvement, with many people still eligible for statin and blood pressure medication but not yet receiving these medicines.

In contrast, obesity had received far less focus for several reasons. Early epidemiological studies did not convincingly identify obesity as a causal risk factor for ASCVD, so it was not included in cardiovascular risk scores until recently. Even then, with few effective treatments available, and a lack of trial evidence to show outcome benefits of weight management in relevant cardiovascular conditions, little attention has been paid to obesity management in the cardiovascular community.

Recently, several factors have led cardiologists to take obesity more seriously. Most strikingly, and somewhat paradoxically, there have been unintended consequences of the medical successes over the past few decades, with fewer cardiovascular deaths and improved survival in people with type 2 diabetes, heart failure, rheumatoid arthritis, and indeed, many cancers [2]. Rising levels of obesity exacerbated by a progressively obesogenic environment, particularly in low-resource communities, have coincided with this longer life expectancy to lead to greater aggregated exposures to excess weight in many, so that average body mass index levels have been increasing in all cardiology clinics.

The consequence is that far more people are now living with multiple long-term conditions, of which many are causally linked to obesity, as we have reviewed [2] (Fig 1). This is because obesity affects so many bodily systems and risk pathways. In particular, it has:

**Fig 1:**
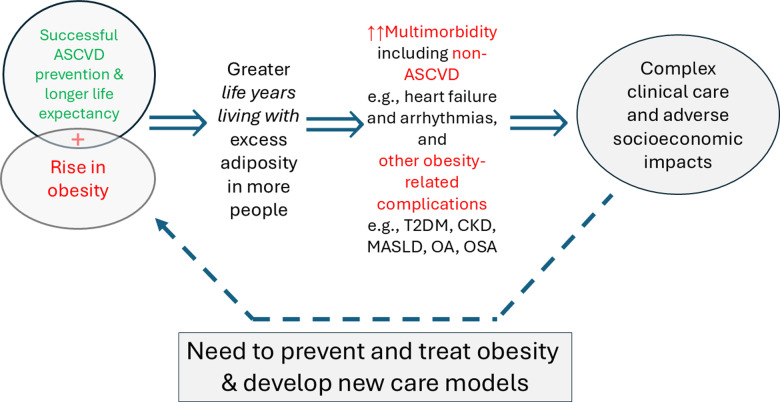
This figure illustrates the convergence of ‘medical success’—successful ASCVD prevention and longer life expectancy in many conditions and in general, allied to the ‘societal failure’ of rising obesity levels in the population. This means more people living progressively longer with more life years with excess adiposity. This pattern, in turn, is (1) promoting rising numbers living with long-term multiple conditions, (2) increasing care complexity and (3) increasing healthcare costs at astonishingly fast rates. There is an urgent need therefore to develop new models of care that are more efficient for patients and doctors alike, and to treat and prevent obesity. ASCVD, atherosclerotic cardiovascular disease; T2DM, type 2 diabetes mellitus; CKD, chronic kidney disease; MASLD, metabolic dysfunction-associated steatotic liver disease; OA, osteoarthritis; OSA, obstructive sleep apnoea.

i) *mechanical effects* on joints and bone due to the physical demands of carrying extra weight, as well as excess fat in the neck, tongue, soft palate and pharyngeal walls contributing to sleep apnoea.
ii) *metabolic effects* often caused by ectopic fat in organs that adversely impact function. For example, ectopic fat in the pancreas, liver and skeletal muscle causes hyperglycaemia and also hypertriglyceridemia, thereby affecting the quality of ‘fuel’ supplied to peripheral organs.
iii) *haemodynamic effects* that increase plasma volume and elevate pressure on organs, contributing to the rising prevalence of heart failure, kidney disease and several other *non-atherosclerotic cardiovascular outcomes* [1] (Fig 1).
iv) *coagulation effects* leading to greater risks for thromboembolic events.
v) *mental health impacts* from the physical limitations of obesity and the associated societal stigma.
vi) *cellular dysfunction* from overnutrition and associated inflammatory stress throughout the body, especially in the heart, liver and kidneys.

This list is far from complete because there is still much to learn about obesity-driven disease mechanisms.

Given the causal role of obesity in many chronic conditions, greater lifetime exposure to adiposity increases the likelihood of developing long-term obesity-related complications [2,3]. This complex disease burden is straining healthcare systems in many high-income countries, particularly in less affluent communities with far lower agency (e.g., education, food deserts, capacity to do sports) to withstand the obesogenic environment, leading to more complex clinical care, rising medical and device costs, and greater distress for patients and their families, including faster loss of independence. At the same time, many low- and middle-income countries are starting to see worrying increases in obesity levels.

From an outsider’s perspective, it may appear that the medical community has been rather slow to recognise and adapt to this changing epidemiology, perhaps due to having ‘disease silo’ perspectives. However, the rise in the number of people living with multiple long-term conditions is stark and obesity is being recognised by healthcare professionals and healthcare policy developers as the primary modifiable contributor. Obesity also reduces economic productivity through higher rates of unemployment and sick leave, thereby compounding adverse economic impacts of obesity driven by higher healthcare costs. This cannot go on.

However, recent health innovations and trials provide some hope for tackling obesity and its consequences, especially in cardiology, bringing cardiologists well and truly into ‘the obesity space’.

First, the semaglutide trials in heart failure with preserved ejection fraction (STEP HFpEF) trials, recently meta-analysed [4], have shown that once-weekly semaglutide (2.4 mg) can help lower body weight, and improve symptoms and functional outcomes in people with heart failure and preserved ejection fraction (HFpEF), regardless of diabetes status. In fact, symptomatic improvement in these trials, measured by the Kansas City Cardiomyopathy Questionnaire, exceeded that associated with sodium-glucose-transporter-2 inhibitor use. Furthermore, these trials, along with the *Studying Multiple Incretins for Heart Failure Treatment* (SUMMIT) trial of tirzepatide in HFpEF [5], have begun to show the potential of anti-obesity medicines (AOMs) to reduce risks for incident heart failure events [4].

Second, the results of the *Semaglutide Effects on Cardiovascular Outcomes in People With Overweight or Obesity* (SELECT) trial [6] have further impressed the Cardiology community. SELECT was the first randomised trial of an AOM (semaglutide 2.4 mg once weekly) to lower risk for major adverse cardiovascular events (MACE), doing so by 20%, in its non-diabetic trial participants with BMI > 27 kg/m^2^ and prior atherosclerotic cardiovascular disease. Those receiving semaglutide not only lost around 10% of their body weight but also felt better and had nominally significant reductions in heart failure outcomes and total mortality, when compared to placebo-treated participants. They also experienced fewer severe adverse events than placebo recipients, despite experiencing higher rates of gastrointestinal side effects. These studies have come off the back of a wealth of evidence, for cardiovascular and more recently, kidney benefits of glucagon-like peptide-1 receptor agonists (GLP-1RAs) in people with type 2 diabetes.

Subsequent papers have shown durable weight loss in those who continue to take these medicines [7] with additional evidence for kidney benefits [8,9], added to anticipated benefits of intentional weight loss on multiple other outcomes. The important point is that, unlike most secondary prevention medicines, semaglutide has potential to not only reduce the risk of MACE in people with obesity but also to improve quality of life by meaningfully reducing weight to an extent that prevents diabetes and benefits other organs systems. This spectrum of benefits is highly appealing to patients.

For these reasons, cardiologists are now paying closer attention to AOMs. Health authorities across the world are likewise starting to approve semaglutide for secondary CVD prevention in people with type 2 diabetes, and relevant reviews in leading cardiology journals encourage their appropriate use [10]. There is ongoing debate over whether the benefits seen in SELECT and the STEP-HpEF trials stem from direct drug effects on vascular tissues or because of weight loss. As recently reviewed [11], trial evidence using lower doses and less potent GLP-1RAs in people with diabetes suggests that MACE risk reduction may be due to direct tissue effects of GLP-1RAs, whereas weight loss probably explains most symptomatic benefits in people with HFpEF.

Regardless, cardiovascular and kidney meetings are dedicating increasing numbers of sessions to obesity, as these specialists join diabetes physicians in acknowledging the interconnectivity of these conditions. This convergence has given rise to a new term of “cardio-kidney metabolic disease.” That said, part of this convergence is also linked to rising life expectancy within each condition so that patients live longer to develop linked conditions due to the enrichment of some common risk factors, with obesity being a major one.

Ongoing outcome trials with AOMs will provide more data on the long-term efficacy and safety of new molecules including dual and triple agonists as well as some containing GIP antagonists [12]. Mechanistic studies are also required to identify molecular pathways to cardio-kidney metabolic outcomes, and the relative contributions of weight loss versus direct drug effects on such pathways.

For now, the high cost of these weight loss drugs limits their widespread use to perhaps only those with life-changing conditions that would benefit from weight loss or those living with several obesity-related comorbidities, and, even when funding is available, demand is outstripping supply in some countries. The hope is that with more such medicines now coming to the market, competition will bring down costs.

However, medication alone cannot solve the obesity epidemic, which now extends to children and women of reproductive age. In addition to newer AOMs, it is essential that we tackle the obesogenic environment in which we live—an immense challenge that no country has successfully addressed.

While we await these developments, and further clinical and mechanistic evidence, the cardiology community, like others, must recognise the impact of obesity on the development and progression of many cardiovascular conditions, now impacting millions of patients worldwide. The cardiology community must also embrace treatments to tackle obesity to help slow the rising incidence of obesity-driven cardiovascular disease and multimorbidity (Fig 1).

## Abbreviations:

AOMs: anti-obesity medicines
ASCVD: atherosclerotic cardiovascular disease
CKD: chronic kidney disease
GLP-1RAs: glucagon-like peptide-1 receptor agonists
HFpEF: heart failure with preserved ejection fraction
MACE: major adverse cardiovascular events
MASLD: metabolic dysfunction-associated steatotic liver disease
OA: osteoarthritis
OSA: obstructive sleep apnoea
SELECT: Semaglutide Effects on Cardiovascular Outcomes in People With Overweight or Obesity
T2DM: type 2 diabetes mellitus
